# Trends and factors associated with modification or discontinuation of the initial antiretroviral regimen during the first year of treatment in the Turkish HIV-TR Cohort, 2011–2017

**DOI:** 10.1186/s12981-020-00328-6

**Published:** 2021-01-09

**Authors:** Volkan Korten, Deniz Gökengin, Gülhan Eren, Taner Yıldırmak, Serap Gencer, Haluk Eraksoy, Dilara Inan, Figen Kaptan, Başak Dokuzoğuz, Ilkay Karaoğlan, Ayşe Willke, Mehmet Gönen, Önder Ergönül, on behalf of the HIV-TR Study Group

**Affiliations:** 1 Department of Infectious Diseases and Clinical Microbiology, Marmara University, Istanbul, Turkey; 2 Department of Infectious Diseases and Clinical Microbiology, Ege University, Izmir, Turkey; 3 Infectious Diseases Clinic, Istanbul Training and Research Hospital, Istanbul, Turkey; 4 Infectious Diseases Clinic, Okmeydanı Training and Research Hospital, Istanbul, Turkey; 5 Infectious Diseases Clinic, Lütfi Kirdar Training and Research Hospital, Istanbul, Turkey; 6 Department of Infectious Diseases and Clinical Microbiology, Istanbul Medical Faculty, Istanbul University, Istanbul, Turkey; 7 Department of Infectious Diseases and Clinical Microbiology, Akdeniz University, Antalya, Turkey; 8 Department of Infectious Diseases and Clinical Microbiology, Izmir Katip Çelebi University, Izmir, Turkey; 9 Infectious Diseases Clinic, Ankara Numune Training and Research Hospital, Ankara, Turkey; 10 Department of Infectious Diseases and Clinical Microbiology, Gaziantep University, Gaziantep, Turkey; 11 Infectious Diseases Clinic, Kent University Academic Hospital, Istanbul, Turkey; 12 College of Engineering and School of Medicine, Koç University, Istanbul, Turkey; 13 Department of Infectious Diseases and Clinical Microbiology, Koç University, Istanbul, Turkey

**Keywords:** Antiretroviral therapy, Treatment modification, Integrase strand transfer inhibitor, Treatment outcome, Cohort study

## Abstract

**Background:**

There is limited evidence on the modification or stopping of antiretroviral therapy (ART) regimens, including novel antiretroviral drugs. The aim of this study was to evaluate the discontinuation of first ART before and after the availability of better tolerated and less complex regimens by comparing the frequency, reasons and associations with patient characteristics.

**Methods:**

A total of 3019 ART-naive patients registered in the HIV-TR cohort who started ART between Jan 2011 and Feb 2017 were studied. Only the first modification within the first year of treatment for each patient was included in the analyses. Reasons were classified as listed in the coded form in the web-based database. Cumulative incidences were analysed using competing risk function and factors associated with discontinuation of the ART regimen were examined using Cox proportional hazards models and Fine-Gray competing risk regression models.

**Results:**

The initial ART regimen was discontinued in 351 out of 3019 eligible patients (11.6%) within the first year. The main reason for discontinuation was intolerance/toxicity (45.0%), followed by treatment simplification (9.7%), patient willingness (7.4%), poor compliance (7.1%), prevention of future toxicities (6.0%), virologic failure (5.4%), and provider preference (5.4%). Non-nucleoside reverse transcriptase inhibitor (NNRTI)-based (aHR = 4.4, [95% CI 3.0–6.4]; p < 0.0001) or protease inhibitor (PI)-based regimens (aHR = 4.3, [95% CI 3.1–6.0]; p < 0.0001) relative to integrase strand transfer inhibitor (InSTI)-based regimens were significantly associated with ART discontinuation. ART initiated at a later period (2015-Feb 2017) (aHR = 0.6, [95% CI 0.4–0.9]; p < 0.0001) was less likely to be discontinued. A lower rate of treatment discontinuation for intolerance/toxicity was observed with InSTI-based regimens (2.0%) than with NNRTI- (6.6%) and PI-based regimens (7.5%) (p < 0.001). The percentage of patients who achieved HIV RNA < 200 copies/mL within 12 months of ART initiation was 91% in the ART discontinued group vs. 94% in the continued group (p > 0.05).

**Conclusion:**

ART discontinuation due to intolerance/toxicity and virologic failure decreased over time. InSTI-based regimens were less likely to be discontinued than PI- and NNRTI-based ART.

## Background

Combination antiretroviral therapy (ART) has significantly reduced the morbidity and mortality of persons living with HIV (PLWH) [1]. The rates of virologic failure with initial ART regimens are decreasing both in clinical trials and in observational cohorts with newer drugs [2, 3]. Discontinuation or modification of the ART regimen is still reported in a minority of patients, especially within the first year, and drug intolerance or toxicity rather than virologic failure is the major reason for discontinuation [4]. Over the past few years, several new drugs with improved efficacy, better tolerability and toxicity profiles, and more convenient dosing and formulations compared to those of historical drugs have become available. Decisions of the healthcare provider and the willingness of the patients may also have contributed to the real-world durability of newer regimens. During the last decade, the majority of new drugs in various ART classes were introduced in Turkey just a few years later than their launch in resource-rich European countries, and they are accessible for PLWH without any restrictions. A few exceptions are single tablet regimens (STRs) containing efavirenz (EFV) or rilpivirine and atazanavir, a commonly used PI in resource-rich countries. Raltegravir (RAL) was not available for first-line treatment until August 2015. Abacavir (ABC) was only available as an STR including dolutegravir (DTG)/ABC/lamivudine (3TC) after October 2016. Providers have usually initiated locally available ART regimens according to the latest United States Department of Health and Human Services (DHHS) or the European AIDS Clinical Society (EACS) guidelines. The first National Guideline for the Management of HIV [5] was published by the Turkish Ministry of Health in late 2013; this guidance included recommendations similar to those in the EACS guidelines released in 2013, and its possible impacts on the choice of ART regimens would be expected to occur after 2014.

Several cohort studies found that various factors might lead to an earlier modification of the initial ART, such as multiple-tablet regimens, more than once daily dosing, injection drug use, treatment with a protease inhibitor (PI) or a high baseline viral load [1, 2, 6–8]. Most of these studies were done before the widespread use of integrase strand transfer inhibitors (InSTI). Randomized controlled trials with novel regimens mainly containing different InSTIs have demonstrated favourable efficacy, tolerability and ease of use [9]. However, studies comparing regimens including older drugs with largely InSTI-based contemporary regimens in terms of durability and reasons for discontinuation of the initial regimen are limited with observational cohorts even in resource rich countries and there is little information available from middle income countries [10–12]. Large-scale studies analysing ART modifications comparing regimens including historical versus novel drugs are not available in Turkey. This study gives us an opportunity to better understand the benefits and disadvantages (if any) of recent regimens. Therefore, the aim of this study was to compare the frequency of as well as the reasons for and factors associated with discontinuation (switching or stopping) of the initial ART regimen among treatment-naive patients before and after the availability of better tolerated and less complex novel regimens.

## Methods

The study protocol was approved by the local ethical review board of the Marmara University School of Medicine (15 Jul 2016, No: 09.2016.398).

This was a retrospective follow-up study conducted within the HIV-TR cohort including 25 tertiary care hospital clinics in 13 cities from different geographic regions in Turkey. The HIV-TR cohort covers approximately one-third of PLWH receiving treatment in Turkey. All treatment-naive adult patients (aged ≥ 18 years) who were registered in the HIV-TR cohort and who started ART between January 1, 2011, and February 28, 2017, were included. Demographic, clinical, laboratory and treatment data extracted from medical records of patients were recorded retrospectively by a web-based data collection system. Patients whose treatment initiation and discontinuation dates were available were eligible for analysis. The outcome was defined as the time to the first modification or stopping of ART during the first year of treatment. Treatment modification was defined as a change in at least one antiretroviral drug in the regimen excluding dose alterations. A stop was defined as the discontinuation of all drugs in the regimen for at least 30 days. The term discontinuation will be used throughout this article for the modification/stopping of treatment because the number of patients stopping ART was few. Reasons for discontinuation were classified as listed in the coded form in the web-based database, including intolerance/toxicity, poor compliance, immunological failure, virologic failure, treatment simplification, drug interactions, pregnancy-related issues, new CDC stage C disease, provider’s decision, patient’s willingness, prevention of future toxicities and others as documented by the clinician. Only the first modification for each patient within 1 year of treatment initiation was included in the analyses. If more than one reason was recorded, the primary reason given by the investigator was included in the analyses. ART regimens were defined according to their classes as follows: 2 nucleoside analogue reverse transcriptase inhibitor (NRTI)s + a 3rd agent [non-nucleoside reverse transcriptase inhibitor (NNRTI), boosted protease inhibitor (PI), or integrase strand transfer inhibitor (InSTI), or an InSTI with a PI (InSTI/PI). NNRTI/InSTI and NNRTI/PI-based regimens were categorized as InSTI-based and PI-based, respectively [10].

The main objective was to analyse and describe the changes in the frequency of and reasons for discontinuation of the initial ART regimens. Patients who died within a year after starting treatment were excluded from the analyses of virologic outcomes. The analysis for the factors associated with treatment discontinuation included the following data: age, gender, transmission risk factor, baseline viral load, pretreatment CD4 count, AIDS diagnosis, ART regimen categories, individual regimens used in patients and ART initiation period. The initiation period was categorized as 2011–2014 and 2015-Feb 2017 according to the year of ART initiation considering the publication of the first national guideline. Frequency and percentages (based on the non-missing data) of observed values were reported for categorical measures. Cumulative incidence curves from competing risk analyses of treatment discontinuation and death were used to describe the cumulative incidence of any cause and intolerance/toxicity-associated first-line regimen discontinuation based on the ART regimen type. The log-rank test was used to compare cumulative incidence curves of different drug classes. We first used Cox regression modelling to assess factors associated with regimen discontinuation. In these analyses, the follow-up time of patients who did not discontinue any drug after the first year of observation was censored at 12 months. Other censor dates were most recent clinic visit for patients who were LTFU or date of discontinuation of ART, whichever occurred first. Death was also treated as a censoring event. Next, we used competing risks regression analysis (Fine and Gray subdistribution hazard model), treating death as a competing risk [13]. All variables associated with discontinuation on bi-variable analysis (p < 0.10) were included in the multivariate analysis by Fine-Gray and Cox models. Independent variables were tested for multiple collinearity before including in Cox model by using the SPSS module. No imputation was done for missing baseline data. We used backward selection eliminating variables to reach the final Cox model. Multivariable models using individual drug regimens or drug classes were separately examined due to their close relationship. Model 2 was used to explore any differences between individual drug regimens in the same class. A sensitivity analysis considering LTFU in the discontinuation group was performed. A separate analysis was also performed to evaluate intolerance/toxicity-associated regimen discontinuation. The TRIPOD guidelines for reporting were followed [14] (Additional file 1: Table **S**1). Chi square and Fisher’s exact tests were used to compare proportions. Mann–Whitney U and Chi square tests were used for comparison of baseline characteristics between groups. SPSS (IBM Corp. Released 2013. IBM SPSS Statistics for Windows, Version 21.0. Armonk, NY) was used for all statistical analyses except for cumulative incidence analysis and Fine and Gray subdistribution hazard model. Competing risks regression analysis was done using the ‘riskRegression’ package in R software, version 2.43-3 (The R Foundation for Statistical Computing, Vienna, Austria). The P value was set at < 0.05 for statistical significance.

## Results

We identified 3019 treatment-naive patients. A total of 122 (4.0%) patients were LTFU within the first year. The median age was 35 years (interquartile range [IQR] 28–45), and the baseline median CD4 cell count was 346 cells/mm^3^ (IQR 196–500). The baseline characteristics of patients and the most common first-line ART regimens chosen within the study period are shown in Table 1.

**Table 1:** Baseline characteristics of patients at treatment initiation by study period

Variable	2011–2014 (n = 1495)	2015–2017 Feb (n = 1524)	P
	N(%)	N(%)	
Male Sex	1242 (83.1)	1363 (89.4)	< 0.001
Age			
Median (IQR), years	37 (29–46)	34 (27–45)	< 0.001
HIV-RNA load			
Median (IQR), log_10_ copies/mL	5.1 (4.6–5.6)	5.1 (4.5–5.7)	0.590
Pretreatment CD4 + cell count			
Median (IQR), cells/µL	325 (166–470)	365 (222–520)	< 0.001
Transmission mode			
MSM/Bisexual	459 (30.7)	579 (38.0)	0.001
Heterosexual	880 (58.9)	803 (52.7)	
IDU	2 (0.1)	4 (0.3)	
Other	36 (2.4)	26 (1.7)	
Unknown	118 (7.9)	112 (7.3)	
Lost to follow up	67 (4.5%)	55 (3.6%)	0.277
Drug Class			
InSTI	24 (1.6)	1111 (72.9)	< 0.001
NNRTI	726 (48.6)	72 (4.7)	
PI	744 (49.8)	336 (22.0)	
InSTI/PI	1 (0.1)	5 (0.3)	
Regimen			
EFV/TDF/FTC	678 (45.4)	72 (4.7)	< 0.001
LPV/r/TDF/FTC	565 (37.8)	178 (11.7)	
EVG/c/TDF/FTC	5 (0.3)	675 (44.3)	
DTG/TDF/FTC	4 (0.3)	347 (22.8)	
DRV/r/TDF/FTC	126 (8.4)	150 (9.8)	
RAL/TDF/FTC	14 (0.9)	51 (3.3)	
LPV/r/ZDV/3TC	47 (3.1)	6 (0.4)	
EFV/ZDV/3TC	39 (2.6)	–	
Other	17 (1.1)	45 (3.0)	

*3TC* lamivudine, *DRV* darunavir, *DTG* dolutegravir, *EFV* efavirenz, *EVG/c* elvitegravir/cobicistat, *FTC* emtricitabine, *InSTI* integrase strand transfer inhibitor, *IDU* injection drug user, *LPV* lopinavir, *MSM* men who have sex with men, *NNRTI* non-nucleoside reverse transcriptase inhibitor, *PI* protease inhibitor, *RAL* raltegravir, *r* ritonavir, *TDF* tenofovir disoproxil fumarate, *ZDV* zidovudine

The median age significantly dropped, and the number of transmissions between men who have sex with men (MSM) increased in the second study period compared to the first. The composition of antiretroviral regimens changed significantly over time, mostly because of the introduction of new drugs [i.e., tenofovir disoproxil fumarate (TDF)/emtricitabine (FTC), darunavir (DRV) and integrase inhibitors (particularly InSTI containing STRs)]. EFV/TDF/FTC and lopinavir (LPV)/ritonavir (r)/TDF/FTC were more commonly prescribed in 2011–2014, while the two InSTI-based regimens (elvitegravir/cobicistat (EVG/c)/TDF/FTC and DTG/TDF/FTC) were more common in the second period (Table 1, Additional file 2: Figure S1). The initial NRTI backbone most commonly included TDF/FTC (95.1%), followed by zidovudine (ZDV)/3TC (3.4%) or ABC-3TC (1.1%).

The initial ART regimen was discontinued in 351 out of 3019 patients (11.6%) within the 12-month follow-up period, with the regimen being modified in 337 (11.2%) and stopped in 14 (0.5%) patients. Twenty-eight patients died before any change in regimen (0.9%). The baseline characteristics of patients who discontinued or continued their ART during the first year are shown in Table 2. Of 337 patients with the initial regimen modified, 6 had a second modification (5 in the early and 1 in the late period). None of the patients had a third modification.

**Table 2:** Characteristics of patients who did or did not discontinue their ART regimen within the first 12 months

Characteristic	Total = 3019	Discontinued = 351	Continued = 2668	P
	N (%)	N (%)	N (%)	
Gender				.083
Male	2605 (86.3)	292 (83.2)	2313 (86.7)	
Female	414 (13.7)	59 (16.8)	355 (13.3)	
Age (years)				*.021*
≤ 45	2240 (75.0)	*244 (69.9)*	*1996 (75.7)*	
> 45	746 (25.0)	*105 (30.1)*	*641 (24.3)*	
Mode of transmission				.598
MSM/Bisexual	1038 (34.4)	115 (32.8)	923 (34.6)	
Heterosexual	1683 (55.7)	198 (56.4)	1485 (55.7)	
IDU	6 (0.2)	1 (0.3)	5 (0.2)	
Other	62 (2.1)	11 (3.1)	51 (1.9)	
Unknown	230 (7.6)	26 (7.4)	204 (7.6)	
Pretreatment CD4 cell count (cells/mm^3^)				*.011*
< 200	723 (25.6)	*104 (31.4)*	*619 (24.8)*	
≥ 200	2103 (74.4)	*227 (68.6)*	*1876 (75.2)*	
Pretreatment viral load (copies/mL)				.213
< 100,000	1262 (45.0)	135 (41.7)	1127 (45.4)	
≥ 100,000	1545 (55.0)	189 (58.3)	1356 (54.6)	
AIDS diagnosis				*.006*
Yes	793 (26.3)	*114 (32.5)*	*679 (25.4)*	
No	2226 (73.7)	*237 (67.5)*	*1989 (74.6)*	
ART regimen type				*<.001*
InSTI	1135 (37.6)	*63 (17.9)*	*1072 (40.2)*	
InSTI/PI	6 (0.2)	*1 (0.3)*	*5 (0.2)*	
NNRTI	798 (26.4)	*109 (31.1)*	*689 (25.8)*	
PI	1080 (35.8)	*178 (50.7)*	*902 (33.8)*	
Type of initial ART				*<.001*
EFV/TDF/FTC	750 (24.8)	*101 (28.8)*	*649 (24.3)*	
LPV/r/TDF/FTC	743 (24.6)	*121 (34.5)*	*622 (23.3)*	
EVG/c/TDF/FTC	680 (22.5)	*39 (11.1)*	*641 (24.0)*	
DTG/TDF/FTC	351 (11.6)	*10 (2.8)*	*341 (12.8)*	
DRV/r/TDF/FTC	276 (9.1)	*34 (9.7)*	*242 (9.1)*	
RAL/TDF/FTC	65 (2.2)	*12 (3.4)*	*53 (2.0)*	
LPV/r/ZDV/3TC	53 (1.8)	*21 (6.0)*	*32 (1.2)*	
EFV/ZDV/3TC	39 (1.3)	*7 (2.0)*	*32 (1.2)*	
Other	62 (2.1)	*6 (1.7)*	*56 (2.1)*	
Year of ART initiation				*.031*
2011–2014	1495 (49.5)	*193 (55.0)*	*1302 (48.8)*	
2015–2017 Feb	1524 (50.5)	*158 (45.0)*	*1366 (51.2)*	

Missing values: 6% for CD4 cell counts, 7% for pretreatment viral load

*3TC* lamivudine, *DRV* darunavir, *DTG* dolutegravir, *EFV* efavirenz, *EVG/c* elvitegravir/cobicistat, *FTC* emtricitabine, *InSTI* integrase strand transfer inhibitor, *IDU* injection drug user, *LPV* lopinavir, *MSM* men who have sex with men, *NNRTI* non-nucleoside reverse transcriptase inhibitor, *PI* protease inhibitor, *RAL* raltegravir, *r* ritonavir, *TDF* tenofovir disoproxil fumarate, *ZDV* zidovudine

The main reason for discontinuation of initial regimen was intolerance/toxicity (45.0%), followed by treatment simplification (9.7%), patient’s willingness (7.4%), poor compliance (7.1%), prevention of future toxicities (6.0%), virologic failure (5.4%), and clinician’s preference (5.4%). The reasons for discontinuation by study period are shown in Table 3. Three of the six patients with a second regimen change were using EFV/TDF/FTC. The reasons for modifications were noted as virologic failure, CNS toxicity due to EFV and poor compliance in each patient. Other second line regimens and reasons for modification were LPV/r/TDF/FTC–gastrointestinal intolerance due to LPV/r, DRV/r/TDF/FTC–hypersensitivity caused by darunavir and RAL/TDF/FTC–nephrotoxicity due to TDF.

**Table 3:** Reasons for ART discontinuation by study period

Reason for discontinuation	Year of initial ART			
	2011–2014 n (%)	2015–Feb 2017 n (%)	Total n (%)	P
Intolerance/toxicity	91 (47.2)	67 (42.4)	158 (45.0)	0.056
Treatment simplification	12 (6.2)	22 (13.9)	34 (9.7)	0.086
Patient’s willingness	16 (8.3)	10 (6.3)	26 (7.4)	0.239
Poor compliance	16 (8.3)	9 (5.7)	25 (7.1)	0.162
Prevention of future toxicities	*6 (3.1)*	*15 (9.5)*	*21 (6.0)*	*0.049*
Virologic failure	*17 (8.8)*	*2 (1.3)*	*19 (5.4)*	*0.001*
Clinician’s preference	6 (3.1)	13 (8.2)	19 (5.4)	0.108
Others	29 (15.0)	20 (12.7)	49 (14.0)	0.199
Total	193 (100)	158 (100)	351 (100)	

p by Chi square

**Fig. 1:**
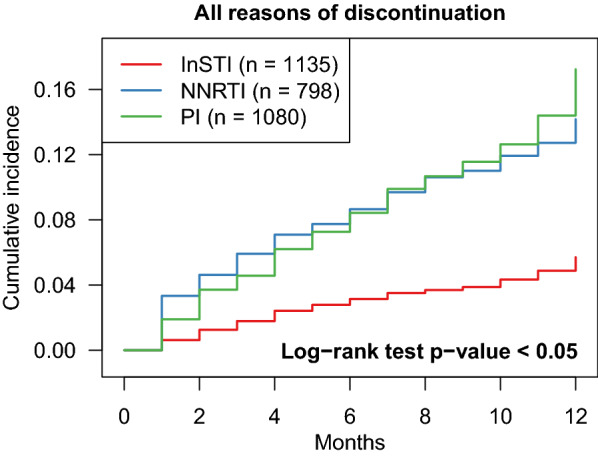
Cumulative incidence curves from competing risk analyses of first regimen discontinuation for all reasons by regimen type

ART discontinuation within the first year of treatment was slightly lower [10.4% (95% confidence interval (CI), 10.0%–12.1%)] during the 2015–Feb 2017 period compared to 2011-2014 [12.9% (95% CI, 11.4%–14.8%)] (Table 2). LTFU rates were similar in two periods (4.5% in the early and 3.6% in the later period, p: 0.277). There was a significant difference in the probability of treatment discontinuation between regimen types (Fig. 1). At 12 months, 16.5% (95% CI 14.5–19.0) of PI-based regimens, 13.7% (95% CI 11.3–16.3) of NNRTI-based regimens, and 5.6% (95% CI 4.3–6.9) of InSTI-based regimens had been discontinued (p < 0.001).

Table 4 presents univariate and multivariable Fine and Gray subdistribution hazard model and Cox proportional hazard models of factors associated with initial ART discontinuation. In the Cox multivariable model 1 including drug class, those initiating ART during the second period (2015-Feb 2017) were less likely to discontinue their regimen within the first year compared to patients initiating ART during 2011-2014 (aHR = 0.6; 95% CI, 0.4– 0.9). Relative to InSTI-based regimens, NNRTI- (aHR = 4.4; 95% CI, 3.0– 6.4) or PI-based (aHR = 4.3; 95% CI, 3.1– 6.0) regimens were significantly more likely to be discontinued. The Fine and Gray model revealed similar subdistribution hazard ratios for the same parameters. In the Cox multivariable model 2, including individual regimens instead of drug classes, initiation period remained significant. Compared to EVG/c/TDF/FTC, other regimens included in the analysis were more likely to be discontinued, excluding DTG/TDF/FTC (Table 4).

**Table 4:** Association of various characteristics with the initial ART discontinuation in treatment-naive patients starting therapy between Jan 2011 and Feb 2017 using competing risk models and cause-specific Cox models

Characteristic	Subdistribution Hazard Model							Cox Proportional Hazard Model					
	Univariate analysis		Multivariable analysis		Multivariable analysis		Univariate analysis			Multivariable analysis		Multivariable analysis	
			Model 1		Model 2					Model 1		Model 2	
	SHR (95% Cl)	*P* value	SHR (95% Cl)	P-value	SHR (95% Cl)	P-value	HR(95% Cl)		P-value	HR (95% Cl)	P-value	HR (95% Cl)	P-value
Age (years)													
≤45	1.0*		1.0*		1.0*		1.0*			1.0*		1.0*	
>45	*1.3 (1.1-1.7)*	*0.014*	1.3 (1.0-1.6)	0.054	1.3 (1.0-1.6)	0.060	*1.3 (1.1-1.7)*		*0.015*	1.3 (1.0-1.6)	0.061	1.2 (1.0-1.6)	0.051
Sex													
Male	1.0*		1.0*		1.0*		1.0*			1.0*		1.0*	
Female	1.3 (1.0-1.7)	0.055	1.2 (0.9-1.6)	0.230	1.1 (0.8-1.4)	0.590	*1.3 (1.0-1.8)*		*0.050*	1.2 (0.9-1.6)	0.202	1.1 (0.8-1.5)	0.634
Transmission mode													
MSM/Bisexual	1.0*						1.0*						
Heterosexual	1.1 (0.9-1.4)	0.520					1.1 (0.9-1.4)		0.432				
IVDU	1.9 (0.3-13.7)	0.530					1.9 (0.3-13.4)		0.532				
Other	1.7 (0.9-3.0)	0.100					1.5 (0.7-2.9)		0.280				
Unknown	1.1 (0.7–1.6)	0.750					1.1 (0.7–1.7)		0.649				
Viral load (copies/mm^3^)													
< 100.000	1.0*						1.0*						
≥ 100.000	1.1 (0.9–1.4)	0.240					1.1 (0.9–1.4)		0.331				
AIDS diagnosis													
Category C or CD4 < 200 cells/mm^3^	*1.4 (1.1–-1.8)*	*0.003*	1.2 (1.0–1.5)	0.110	1.2 (0.9–1.5)	0.200	*1.4 (1.1–1.8)*		*0.001*	1.3 (1.0–1.6)	0.056	1.2 (1.0–1.5)	0.136
Initiation era													
2011–2014	1.0*		1.0*		1.0*		1.0*			1.0*		1.0*	
2015–2017(Feb)	*0.8 (0.6–1.0)*	*0.021*	*0.5 (0.4–0.7)*	*<.0001*	*0.4 (0.3–0.6)*	*<.0001*	*0.8 (0.6–1.0)*		*0.023*	*0.6 (0.4–0.9)*	*<.0001*	*0.5 (0.3–0.6)*	*<.0001*
Class													
InSTI	1.0*		1.0*				1.0*			1.0*			
NNRTI	*2.6 (1.9–3.6)*	*<.0001*	*4.4 (3.0–6.6)*	*<.0001*			*2.6 (1.9–3.6)*		*<.0001*	*4.4 (3.0–6.4)*	*<.0001*		
PI	*3.2 (2.4–4.2)*	*<.0001*	*4.4 (3.2–6.1)*	*<.0001*			*3.2 (2.4–4.2)*		*<.0001*	*4.3 (3.1–6.0)*	*<.0001*		
Regimen													
EVG/c/TDF/FTC	1.0*				1.0*		1.0*					1.0*	
EFV/TDF/FTC	*2.5 (1.7–3.7)*	*<.001*			*4.8 (3.1–7.5)*	*<.0001*	*2.5 (1.8–3.7)*		*<.0001*			*4.8 (3.1–7.3)*	*<.0001*
LPV/r/TDF/FTC	*3.0 (2.1–4.3)*	*<.001*			*4.9 (3.3–7.3)*	*<.0001*	*3.1 (2.1–4.4)*		*<.0001*			*4.9 (3.3–7.3)*	*<.0001*
DTG/TDF/FTC	*0.5 (0.2–1.0)*	*0.048*			*0.5 (0.2–0.9)*	*0.032*	0.5 (0.3–1.0)		0.053			0.5 (0.2–0.9)	0.054
DRV/r/TDF/FTC	*2.3 (1.4–3.6)*	*<.001*			*2.8 (1.8–4.5)*	*<.0001*	*2.3 (1.4–3.6)*		*0.001*			*2.8 (1.8–4.5)*	*<.0001*
RAL/TDF/FTC	*3.4 (1.8–6.6)*	*<.001*			*3.5 (1.8–6.7)*	*<.0001*	*3.4 (1.8–6.6)*		*<.0001*			*3.5 (1.8–6.7)*	*<.0001*
LPV/r/ZDV/3TC	*9.0 (5.3–15.2)*	*<.001*			*15.5 (8.5–28.4)*	*<.0001*	*9.5 (5.6–16.2)*		*<.0001*			*16.2 (8.9–29.4)*	*<.0001*
EFV/ZDV/3TC	*3.3 (1.5–7.3)*	*0.003*			*6.6 (2.8–15.3)*	*<.0001*	*3.3 (1.5–7.4)*		*0.004*			*6.4 (2.8–15.1)*	*<.0001*
Other	1.6 (0.6–4.0)	0.350			1.8 (0.7–4.6)	0.240	1.6 (0.6–4.1)		0.323			1.8 (0.7–4.5)	0.228

*sHR* subdistribution hazard ratio, *HR* hazard ratio, *CI* confidence interval, *3TC* lamivudine, *DRV* darunavir, *DTG* dolutegravir, *EFV* efavirenz; *EVG/c* elvitegravir/cobicistat, *FTC* emtricitabine, *InSTI* integrase strand transfer inhibitor, *IDU* injection drug user, *LPV* lopinavir, *MSM* men who have sex with men, *NNRTI* non-nucleoside reverse transcriptase inhibitor, *PI* protease inhibitor, *RAL* raltegravir, *r* ritonavir, *TDF* tenofovir disoproxil fumarate, *ZDV* zidovudine

Model 1 includes common displayed variables and drug classes (excluding InSTI/PI)

Model 2 includes common displayed variables and most common regimens

*is reference category

However, patients receiving InSTI-based regimens had less severe disease, indicated by fewer baseline AIDS diagnoses and lower HIV RNA levels than those on PI-based and fewer baseline AIDS diagnoses than those on NNRTI-based regimens. Similarly, those on InSTI-based STRs had fewer baseline AIDS diagnoses but similar HIV RNA levels compared to those on non-STR InSTI-based regimens (Additional file 3: Table S2 and Additional file 4: Table S3). Among patients who modified their treatment, the substitution usually included drugs within the same class (74.6%) in the InSTI-based group and from another class (80.7% and 67.4%) in the NNRTI and PI groups, respectively. In a sensitivity analysis, when patients who were LTFU were included in the outcome group with other discontinuation reasons, same factors remained significant in the model.

**Fig. 2:**
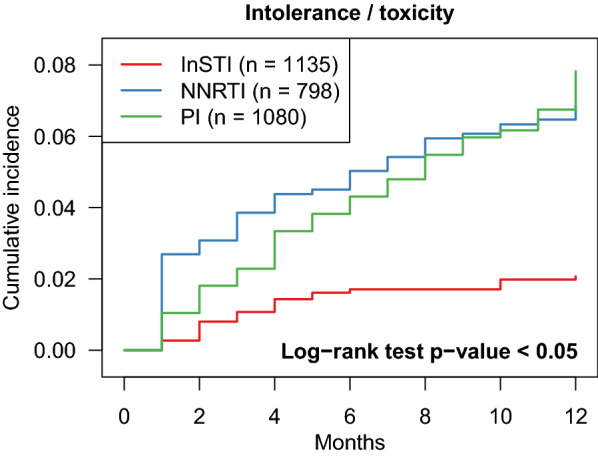
Cumulative incidence curves from competing risk analyses of first regimen discontinuation for intolerance/toxicity by regimen type

The rate of treatment discontinuation for intolerance/toxicity was lower with InSTI-based regimens (2.0% [95% CI 1.2–2.9]) than with NNRTI-based regimens (6.6% [95% CI 5.0–8.3]) and PI-based regimens (7.5% [95% CI 6.0–9.2]) (p < 0.001) (Fig. 2).

Both multivariable Cox models revealed that age > 45 years, early ART initiation period (2011–2014) and non-InSTI regimens were associated with intolerance/toxicity-related discontinuation (Table 5).

**Table 5:** Association of various characteristics with intolerance/toxicity-related ART discontinuation by Cox proportional hazard models in naive patients starting therapy between Jan 2011 and Feb 2017

Characteristic	Univariate analysis		Multivariable analysis		Multivariable analysis	
			Model 1		Model 2	
	Hazard Ratio (95% Cl)	P-value	Hazard Ratio (95% Cl)	P-value	Hazard Ratio (95% Cl)	P-value
Age(years)						
≤ 45	1.0		1.0		1.0	
> 45	*1.5 (1.1–2.1)*	*0.018*	*1.4 (1.0–2.0)*	*0.036*	*1.4 (1.0–2.0)*	*0.044*
Sex						
Male	1.0		1.0		1.0	
Female	*1.6 (1.1–2.3)*	*0.022*	1.4 (1.0–2.1)	0.089	1.3 (1.0–2.0)	0.189
Transmission mode						
MSM/Bisexual	1.0*					
Heterosexual	1.1 (0.8–1.6)	0.519				
IVDU	1.3 (0.2–2.0)	0.960				
Other	1.0 (0.3–3.3)	0.964				
Unknown	1.3 (0.7–2.4)	0.341				
Viral load (copies/mm^3^)						
< 100.000	1.0					
≥ 100.000	1.0 (0.7–1.4)	0.936				
AIDS diagnosis						
Category C or CD4 < 200 cells/mm^3^	1.3 (0.9–1.8)	0.141				
Initiation era						
2011–2014	1.0		1.0		1.0	
2015–2017 Feb	*0.7 (0.5–1.0)*	*0.024*	*0.5 (0.4–0.9)*	*0.001*	*0.5 (0.3–0.8)*	*<.0001*
Class						
InSTI	1.0*		1.0*			
NNRTI	*3.5 (2.1–5.7)*	*<.0001*	*5.8 (3.3–10.5)*	*<.0001*		
PI	*3.9 (2.5-6.3)*	*<.0001*	*5.6 (3.4–9.3)*	*<.0001*		
Regimen						
EVG/c/TDF/FTC	1.0*				1.0*	
EFV/TDF/FTC	*4.0 (2.1–7.6)*	*<.0001*			*7.6 (3.7–15.3)*	*<.0001*
LPV/r/TDF/FTC	*4.6 (2.5–8.6)*	*<.0001*			*7.5 (3.8–14.6)*	*<.0001*
DTG/TDF/FTC	1.3 (0.5–3.2)	0.556			1.2 (0.5–3.0)	0.667
DRV/r/TDF/FTC	*3.4 (1.6–7.3)*	*0.001*			*4.4 (2.0–9.3)*	*<.0001*
RAL/TDF/FTC	1.8 (0.4–8.2)	0.422			1.8 (0.4–8.2)	0.430
LPV/r/ZDV/3TC	*11.3 (4.6–27.6)*	*<.0001*			*19.3 (7.4–50.6)*	*<.0001*
EFV/ZDV/3TC	*4.5 (1.3–16.0)*	*0.020*			*8.6 (2.3–32.4)*	*0.001*
Other	1.0 (0.1–8.0)	0971			*1.2 (0.2-8.9)*	*0890*

*3TC* lamivudine, *DRV* darunavir, *DTG* dolutegravir, *EFV* efavirenz, EVG/c elvitegravir/cobicistat, *FTC* emtricitabine, *InSTI* integrase strand transfer inhibitor, *IDU* injection drug user, *LPV* lopinavir, *MSM* men who have sex with men, *NNRTI* non-nucleoside reverse transcriptase inhibitor, *PI* protease inhibitor, *RAL* raltegravir, *r* ritonavir, *TDF* tenofovir disoproxil fumarate, *ZDV* zidovudine

Model 1 includes common displayed variables and drug classes (excluding InSTI/PI)

Model 2 includes common displayed variables and most common regimens

*is reference category

The most commonly prescribed NNRTI- and PI-based regimens during the study period were significantly more likely to be discontinued for intolerance/toxicity compared to InSTI based regimen-elvitegravir/cobicistat/tenofovir disoproxil fumarate/emtricitabine (EVG/c/TDF/FTC). However, there was no significant difference between EVG/c/TDF/FTC and other InSTI-based regimens, including dolutegravir/tenofovir disoproxil fumarate/emtricitabine (DTG/TDF/FTC) and raltegravir/tenofovir disoproxil fumarate/emtricitabine (RAL/TDF/FTC) (Table 5). While any-cause discontinuation was more likely with RAL/TDF/FTC compared to other InSTI-based regimens, intolerance/toxicity-related discontinuation was similar between regimens, suggesting other reasons leading to the outcome. The DTG/ABC/3TC STR was introduced in the last year of the study, and the interpretation of the regimen analysis for this combination was difficult due to the short follow-up time. NRTIs were considered to be responsible for discontinuation due to intolerance/toxicity in 1.2% of InSTI-based regimens (95% CI 0.64–1.96), in 0.4% of NNRTI-based regimens (95% CI 0.01–0.97) and 1.9% of PI-based regimens (95% CI 0.94–2.48). The distribution of toxicities reflected the established side effects of the drugs (mainly central nervous system side effects and rash for NNRTIs and gastrointestinal intolerance and hyperlipidaemia for PIs).

The rate of discontinuation for intolerance/toxicity decreased over time (6.1% for 2011–2014 vs. 4.4% for 2015–Feb 2017), while modification for treatment simplification displayed an increasing trend (0.8% and 1.4% during 2011–2014 and 2015–2017 Feb, respectively). Virologic failure was not commonly reported as a reason for treatment change and decreased over time significantly (Table 3). In the later period, proactive change of regimen for prevention of future toxicities increased significantly (Table 3). The percentages of patients who achieved HIV RNA levels of < 50 and < 200 copies/mL within 12 months of ART initiation were 85% and 91% in the ART discontinued group vs. 87% and 94% in the continued group, respectively (p > 0.05).

## Discussion

This retrospective cohort analysis examined the trend and factors associated with discontinuation of the initial antiretroviral regimen during the first year of antiretroviral therapy in the Turkish HIV-TR cohort from 2011 to 2017. The cumulative incidence of and reasons for regimen discontinuation were described. The results suggest a decline in all-cause and intolerance/toxicity-associated regimen discontinuation over the study period in the cohort. There was a significant rise in the median CD4 cell count at ART initiation, which reflects the recent changes in the recommendations of major guidelines for initiation of ART regardless of CD4 count [15]. On the other hand, the decreasing median age and the increasing frequency of MSM patients during the study period is in line with the current epidemiologic data for the country [16].

The 12-month probability of continuing initial ART regimens was similar over time, with a slight increase during the second period (from 81.4% in 2011–2014 to 85.4% in 2015–Feb 2017). Overall, drug intolerance/toxicity was the main reason for discontinuation of first-line ART, consistent with many other reports [2, 6, 17]. There was a dramatic shift in prescribing patterns over time, with InSTI-based regimens rapidly replacing PI- and NNRTI-based regimens during the second period of the study. The decreasing rate of discontinuations due to intolerance/toxicity is in line with the introduction of InSTIs; compared to NNRTIs and PIs, InSTI-based regimens were least likely to be discontinued due to intolerance/toxicity, which may be attributed to their favourable toxicity profiles. Another significant finding in the study was the decreasing rate of discontinuations due to virologic failure over time (8.8% in 2011–2014 to 1.3% in 2015–Feb 2017). Although patients initiating InSTI-based regimens had less severe disease (higher CD4 counts and lower viral loads) compared to those using PI- and NNRTI-based regimens, most likely due to the trend to initiate ART regardless of CD4 count, the differences in toxicity/intolerance-related discontinuations suggest that the recent introduction of these convenient, well-tolerated regimens has resulted in better adherence, leading to fewer virologic failures. The increasing trend in discontinuations due to regimen simplification and/or convenience or provider-initiated discontinuations during the later period also suggests a better tolerability profile and dosing convenience of newer regimens. Several studies showed a better durability of once-daily, especially STR [11, 18]. Although INSTI-based regimens showed less discontinuation compared to other classes, we could not find any difference between single or multi-tablet once daily regimens in the class.

The median duration of first-line ART regimens is variable in different settings depending on geographic area and income, treatment periods and availability of drugs. In the past, particularly in resource-rich settings, the probability of treatment change during the first year of treatment was high. The Swiss cohort study reported 37.0% and 45.6% treatment modifications among treatment-naive patients during 1995–1998 and 2000–2005, respectively [2]. The Italian ICONA cohort reported a 36.1% one-year probability of discontinuation of at least 1 drug in the initial regimen during 1997–2007 [6]. In the US HIV Outpatient Study (HOPS), the rates of treatment change or discontinuation gradually decreased from 53.0% during 1996–1999 to 34.5% during 2008–2011 [19]. The Antiretroviral Therapy Cohort Collaboration (ART-CC) cohort reported 25% treatment modification within the first year of treatment (2002–2009) [20]. On the other hand, the rates of treatment modifications are much lower in resource-limited settings; 16.1% of PLWH in China (during 2005–2013) [17], 19% in Ethiopia (during 2010–2014) [21] and 16.3–12.1% in the Asia–Pacific region (during 2003–2013) [22] were not taking their initial regimens at the end of 12 months. Similar rates of switching or stopping (13.3 per 100 person years, 24.9% in the first year) were reported by Kenya (2006–2007) [23]. A large-scale cohort analysis from resource limited countries in sub-Saharan Africa revealed that rates of switching to second-line ART were very low (1.63 per 100 person-years [95% CI 1.60–1.66]) in the absence of CD4 count and viral load measurements [24]. A study from middle-income Latin American and the Caribbean countries covering 2000–2014 also reports a lower rate of virologic failure or major regimen change (12.1%) after one year of ART in 6 countries compared to those in Europe and North America [25]. In resource-limited countries with lower discontinuation rates, switching of the ART regimen is mainly driven by virologic failure [24], and limited alternative treatment options do not allow a switch for patients experiencing intolerance/toxicity [22]. Our results suggest a lower rate of modification of first-line ART compared to the rates reported in earlier observational cohort studies from resource-rich countries while similar rates were observed compared to those from middle income countries [22, 25].

The decision by the patient or the healthcare provider to stop or change an ART regimen will depend to some extent on the availability of alternatives. Although many drugs in major ART classes were available and accessible without any restrictions in Turkey throughout the study period, non-availability of some drugs and formulations in the earlier periods might have been a reason to prevent modification due to toxicity/intolerance in patients with mild side effects. Because we studied only the initial modifications of treatment within the first year, the role of new drug availability on the patient’s willingness and providers’ preference was expected to be limited and contributed to the durability in the last period of the study. Thus, our results are mostly valid for the time period studied in our country and may not be applicable to other countries. Recent studies from Germany reported that most changes within the first year were not driven by virologic failure or adverse events, but were strategic such as preventing future toxicities with increasing options of modern ART [11, 12]. We noted that strategic treatment changes also increased in Turkey despite being a middle-income country in the late period of the study with the unrestricted availability of new drugs.

Various studies have reported that gender, age, transmission mode, treatment period, specific drugs or ART regimen may be major factors for discontinuation of first-line ART regimens [2, 10, 22]. Rates of ART discontinuation were found to be remarkably higher for injection drug users (IDUs) and/or HIV/hepatitis C virus (HCV) co-infected patients compared to other groups in many studies [6, 20, 26–28]. It is difficult to comment on this finding for our study because the number of IDUs and HCV co-infected patients was negligible in our cohort. Several previous studies have found higher rates of discontinuation among older patients, most likely due to co-medications or comorbidities [4, 29]. Age was associated with intolerance/toxicity-related ART discontinuation in both models of multivariate analysis in our study. Severe immunodeficiency and/or HIV-related conditions prior to the initiation of ART, reflected by low CD4 counts or AIDS diagnosis, were associated with higher rates of discontinuation of first-line ART regimens in several previous reports [2, 17, 19, 20, 22, 29]. Patients with other HIV-related conditions that may require co-medications that can interact with the drugs in the ART regimen may experience treatment modification more frequently than those without. Although AIDS diagnosis was associated with an increased probability of treatment discontinuation in the bivariate analysis, this difference was not statistically significant in the multivariate analysis.

This study is limited by lack of data on the level of adherence and comorbidities influencing regimen durability. On the other hand, the results may be generalized to the country level because the vast majority of geographic regions were represented in the study. In addition, it was possible to assess almost all reasons for ART discontinuations because the database included a specific section for that. The HIV prevalence is low in Turkey, and care of PLWH is linked to infectious disease units, where drugs are freely available for the vast majority of patients. When a patient is linked to care, retention in care is high, as reflected by the low rates of LTFU in this study. Very few patients are IDUs or are co-infected with HCV. All of this might have contributed to the favourable outcomes in this study regardless of treatment discontinuation. On the other hand, the observational design of our study resulted in differences for baseline characteristics of patients and treatment options in two study periods. Prescribing patterns and provider preferences may change over time. Therefore, unmeasured factors may be associated with regimen discontinuation in two different time periods in our study.

In conclusion, similar rates of ART discontinuation in the first year were observed in our cohort compared to those from other middle-income countries. This study suggests that all-cause discontinuation and discontinuation due to intolerance/toxicity within the first year of ART have decreased over time, most likely associated with the better tolerability and dosing convenience of newer, mostly InSTI-based regimens. Non-InSTI-based regimens, the early ART initiation period (2011–2014) and older patients (> 45 years old) were associated with a higher risk of ART discontinuation. Our results suggest that the treatment discontinuations were well managed and did not lead to poor virologic outcomes.

## Supplementary information

**Additional file 1: Table S1.** TRIPOD Checklist: Prediction Model Development and Validation.

**Additional file 2: Figure S1.** Prescribing patterns for initial ART in the HIV-TR cohort between 2011 and Feb 2017.

**Additional file 3: Table S2.** Pretreatment virological and immunological characteristics of patients receiving InSTI-, PI- and NNRTI-based regimens.

**Additional file 4: Table S3.** Pretreatment characteristics of patients receiving STR and non-STR InSTI.

## Data Availability

The datasets generated during and/or analysed during the current study are available in the Mendeley repository, https://data.mendeley.com/datasets/p49jpw8khk/1.
