# Supplementary figures and images for: Trends and factors associated with modification or discontinuation of the initial antiretroviral regimen during the first year of treatment in the Turkish HIV-TR Cohort, 2011–2017
Source: AIDS Res Ther. 2021 Jan 9;18:4. doi: 10.1186/s12981-020-00328-6 (PMC7796577; doi:10.1186/s12981-020-00328-6)

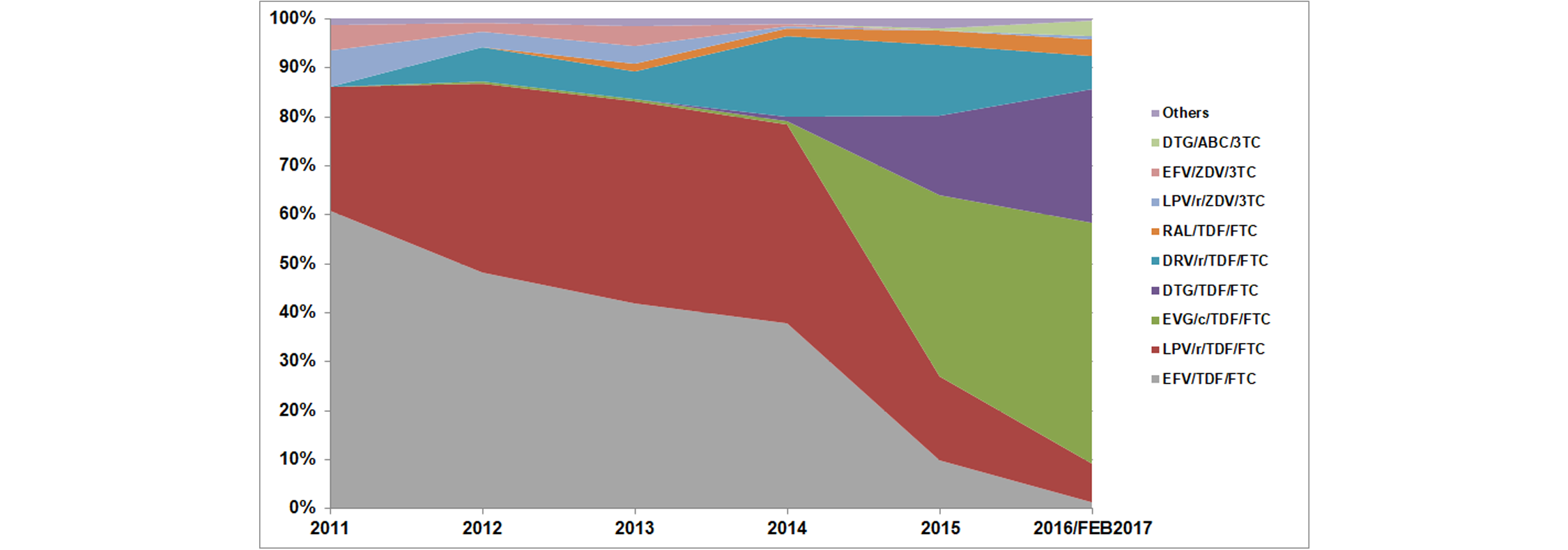

Supplement: Supplementary file 2 — Additional file 2: Figure S1. Prescribing patterns for initial ART in the HIV-TR cohort between 2011 and Feb 2017. [file 12981_2020_328_MOESM2_ESM.tiff]
