# Supplementary material for: Trends and factors associated with modification or discontinuation of the initial antiretroviral regimen during the first year of treatment in the Turkish HIV-TR Cohort, 2011–2017
Source: AIDS Res Ther. 2021 Jan 9;18:4. doi: 10.1186/s12981-020-00328-6 (PMC7796577; doi:10.1186/s12981-020-00328-6)
Supplement: Supplementary file 3 — Additional file 3: Table S2. Pretreatment virological and immunological characteristics of patients receiving InSTI-, PI- and NNRTI-based regimens. [file 12981_2020_328_MOESM3_ESM.docx]

Supplemental Table 2. Pretreatment virological and immunological characteristics of patients receiving InSTI-, PI- and NNRTI-based regimens.

| Characteristic | InSTI | PI | NNRTI | p |
| --- | --- | --- | --- | --- |
| CD4 cell count (mm^3^) at regimen initiation |  |  |  | <.001* |
| <200 | 193 (18.1) | 348 (34.7) | 182 (24.3) | .001** |
| ≥200 | 876 (81.9) | 654 (65.3) | 568 (75.7) |  |
| Category C or CD4<200 |  |  |  | <.001* |
| Yes | 219 (19.3) | 374 (34.6) | 198 (24.8) | .001** |
| No | 916 (80.7) | 706 (65.4) | 600 (75.2) |  |
| Viral load at regimen initiation (copies per mL) |  |  |  | .011* |
| <100.000 | 512 (47.7) | 414 (41.9) | 334 (45.1) | .206*** |
| ≥100.000 | 561 (52.3) | 575 (58.1) | 406 (54.9) |  |
| Reasons for treatment change (Intolerance/ Toxicity) | 23 (2.0) | 81 (7.5) | 53 (6.6) | <.001* |
|  |  |  |  | .001** |

* p<0.05 for InSTI vs PI.

** p<0.05 for InSTI vs NNRTI.

*** p>0.05 for InSTI vs NNRTI.
