# Supplementary material for: Trends and factors associated with modification or discontinuation of the initial antiretroviral regimen during the first year of treatment in the Turkish HIV-TR Cohort, 2011–2017
Source: AIDS Res Ther. 2021 Jan 9;18:4. doi: 10.1186/s12981-020-00328-6 (PMC7796577; doi:10.1186/s12981-020-00328-6)
Supplement: Supplementary file 4 — Additional file 4: Table S3. Pretreatment characteristics of patients receiving STR and non-STR InSTI. [file 12981_2020_328_MOESM4_ESM.docx]

**Supplemental Table 3.** Pretreatment characteristics of patients receiving STR and non-STR InSTI

| Characteristic | STR InSTI | Non-STR InSTI | p |
| --- | --- | --- | --- |
| CD4 cell count (mm^3^) at regimen initiation |  |  | <.001 |
| <200 | 86 (12.8) | 107 (26.8) |  |
| ≥200 | 584 (87.2) | 292 (73.2) |  |
| Category C or CD4<200 |  |  | <.001 |
| Yes | 97 (13.7) | 122 (28.6) |  |
| No | 612 (86.3) | 304 (71.4) |  |
| Viral load at regimen initiation (copies per mL) |  |  | 0.344 |
| <100.000 | 327 (48.9) | 185 (45.8) |  |
| ≥100.000 | 342 (51.1) | 219 (54.2) |  |
| Reasons for treatment change (Intolerance/ Toxicity) | 13 (1.8) | 10 (2.3) | 0.348 |
